# Current Insights into the Role of UV Radiation-Induced Oxidative Stress in Melanoma Pathogenesis

**DOI:** 10.3390/ijms252111651

**Published:** 2024-10-30

**Authors:** Ernest Gieniusz, Elżbieta Skrzydlewska, Wojciech Łuczaj

**Affiliations:** Department of Analytical Chemistry, Medical University of Bialystok, Mickiewicza 2D, 15-222 Bialystok, Poland; 38694@student.umb.edu.pl (E.G.); elzbieta.skrzydlewska@umb.edu.pl (E.S.)

**Keywords:** cellular signaling, melanocytes, melanoma, melanoma progression, melanoma metastasis, molecular background, oxidative stress, UV radiation

## Abstract

Cutaneous melanoma accounts for the majority of skin cancer-related deaths, and its incidence increases each year. The growing number of melanoma cases, especially in advanced stages, poses a significant socio-medical challenge throughout the world. Extensive research on melanoma pathogenesis identifies UV radiation as the most important factor in melanocytic transformation. Oxidative effects of UV irradiation exert their influence on melanoma pathogenesis primarily through modification of nucleic acids, proteins, and lipids, further disrupting cellular signaling and cell cycle regulation. Its effects extend beyond melanocytes, leading to immunosuppression in the exposed skin tissue, which consequently creates conditions for immune surveillance evasion and further progression. In this review, we focus on the specific molecular changes observed in the UV-dependent oxidative stress environment and their biological consequences in the course of the disease, which have not been considered in previous reviews on melanoma. Nonetheless, data show that the exact role of oxidative stress in melanoma initiation and progression remains unclear, as it affects cancerous cells differently depending on the specific context. A better understanding of the pathophysiological basis of melanoma development holds promise for identifying potential targets, which could lead to effective melanoma prevention strategies.

## 1. Introduction

Cutaneous melanoma is a malignancy arising from melanocytes, pigment-producing cells located in basilar epidermis [1]. It is the most aggressive type of skin cancer characterized by rapid progression and substantial metastatic potential [2]. The prevalence of melanoma is higher among fair-skinned individuals [3], and there are many recognized risk factors, including exposure to UV radiation, immunosuppression, and the presence of multiple nevi, especially dysplastic nevi [4,5]. In 2020 alone, over 57,000 patients died from melanoma, accounting for 80% of all skin cancer deaths [6]. Despite numerous screening programs, the global melanoma incidence reached 325,000 new cases in 2020 and is increasing every year, posing a significant challenge for healthcare systems in developed countries [7]. Moreover, melanoma patients from emerging nations tend to have a worse prognosis due to limited access to medical assistance and late diagnoses [8].

Melanoma development is initiated by biological changes in melanocytes undergoing neoplastic transformation. Melanocytes, dendritic pigment cells originating from the neural crest during embryonic development, are ubiquitously distributed throughout the human body, found not only in the skin but also in sites such as the eyes, heart vessels, leptomeninges, and inner ear [9]. These cells, known for their primary pigmentation function and subsequent protection against UV radiation, are also involved in antimicrobial defense and innate immunity [10]. Although the most common location of melanoma is the skin, melanomas also arise in areas such as the mucosal membranes (mucosal melanoma) and eyes (uveal melanoma, conjuctivital melanoma). Ocular melanoma, especially the subtype uveal melanoma, is the foremost intraocular malignancy in adults, with risk factors ranging from congenital ocular melanocytosis to the suggested influence of UV radiation [11]. These ocular melanomas differ from cutaneous melanomas in that they are characterized by rare familial relationships and different biological behaviors [12]. Mucosal melanomas, although less frequent, tend to be more aggressive, often diagnosed late due to their hidden anatomical presence [13]. These melanomas can arise from a wide range of mucosal surfaces, including the nasal cavity, sinuses, oral cavity, anorectum, and various parts of the respiratory, gastrointestinal, and urogenital tracts [14]. Another form of this cancer are subungual melanomas, characterized by a poorer prognosis, metastatic tendency, and debated etiology [15].

Although melanoma can be located in various places in the human body, the vast majority of melanomas are cutaneous [16]. There are four main histological types of skin melanomas: superficial spreading melanoma (SSM), lentigo maligna melanoma (LMM), acral lentiginous melanoma (ALM), desmoplastic melanoma (DM), and nodular melanoma (NM) [17], with SSM being the most common one [18]. Research shows that histological type affects melanoma patients’ prognosis [19], and frequencies of certain genetic mutations differ depending on melanoma subtype, further influencing diverse molecular pathways in tumor cells [20]. Assessment of melanoma pathogenesis, expressed by altered cellular signaling in response to carcinogenic factors, plays a vital role in the advancement of early detection, prevention, and targeted melanoma treatment [21]. Therefore, a thorough understanding of skin melanoma formation mechanisms is crucial for managing this worldwide health problem. Hopefully, new findings in this field may also positively influence extracutaneous melanoma patients’ prognosis.

So far, research on the cause of melanoma development has focused primarily on the impact of sunlight reaching human skin and its action as the main initiator of this cancer. Prolonged exposure of the skin to sunlight results in the disturbance of redox balance in skin cells, including melanocytes, inducing mechanisms leading to the cancer development. Therefore, the purpose of this review is to systematize the knowledge regarding the contribution of UV radiation and oxidative stress in the formation and development of melanoma based on the results of recent studies.

## 2. Melanoma Epidemiology and Classification

Current data indicates that the main factors driving the development and progression of melanoma, in addition to inherited genetic predispositions, are changes in the DNA structure of melanocytes induced by UV radiation [22]. Therefore, melanoma can be classified according to location and duration of exposure to sunlight as high chronic sun damage (high CSD), low chronic sun damage (low CSD), and non-solar subtypes (Table 1).

Further classification extends beyond anatomical location, as each group represents distinct carcinogenic pathways with distinctive mutations and biology. However, due to the widespread nature of sunlight, this review mainly concerns the pathogenesis of cancer resulting from the influence of ultraviolet radiation. High-CSD type includes LMM and DM cancers occurring mainly on the skin of the head, neck, and distal surface of extremities [17,23]. Microscopic examination of the surrounding tissue often reveals signs of prolonged UV exposure, such as solar elastosis [17]. In this specific type of melanoma, the patient demographic typically comprises individuals aged 55 and older with a low nevi count. Notably, tumor alterations in this variant exhibit a heightened frequency of mutations within the cell cycle regulating genes like neurofibromin 1 (NF-1), proto-oncogene tyrosine-protein kinase (ROS-1), neuroblastoma RAS viral oncogene homolog (NRAS), tyrosine-protein kinase KIT (KIT), and v-Raf murine sarcoma viral oncogene homolog B (BRAF) [23]. On the contrary, low-CSD melanomas are mostly represented by the SSM histological subtype, affecting areas sporadically exposed to sunlight, namely the trunk and proximal parts of extremities [23]. Patients diagnosed with this variant are younger, generally under the age of 55. Mutation burden associated with the development of low-CSD melanoma is lower in comparison to that of the high-CSD type, consisting mainly of BRAF V600E mutations [23].

In light of the overwhelming epidemiological evidence indicating the increased melanoma risk associated with excessive sunlight exposure, particularly to the UV part of its spectrum, the use of cosmetics with sun protection filters (SPF) represents a rational strategy for the prevention of melanoma. These products, varying in chemical composition, either reflect or absorb UV rays, protecting the skin, including melanocytes, from the adverse effects of irradiation, such as oxidative stress, inflammation, and photoaging [28,29]. However, despite promising research outcomes in the past, recent trends indicate a positive correlation between sunscreen use and the incidence of melanoma [30]. This paradox represents a multi-level problem, with the potential causes including the increased detection of cases of the disease, the use of insufficient amounts of formulations with SPF, including lack of reapplication, and overexposure to sunlight through a false impression of effective protection [30,31].

The risk of developing melanoma does not only depend on sun exposure; a metaanalysis by An. et al. revealed that the use of indoor tanning beds increases the risk of developing skin cancer, particularly early-onset melanoma [32]. Consequently, there is an ongoing need to educate the public about the consequences of overexposure to ultraviolet radiation, including outdoor and indoor tanning, and the proper use of sunscreen formulations. This is evidenced by a recent survey on the state of knowledge about skin cancer prevention among melanoma patients, revealing a lack of awareness regarding these issues, which may have contributed to the development of the disease in the studied group [33].

## 3. The Most Important Molecular Pathways in Melanomagenesis Affected by UV Radiation

**Figure 1:**
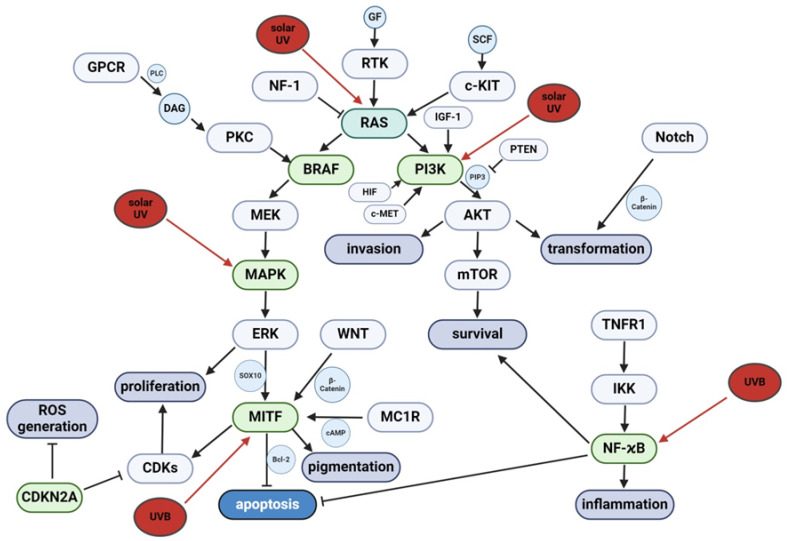
The most important molecular signaling pathways activated by UV irradiation and their involvement in the pathogenesis of melanoma [34,35,36,37,40,41,42,43,44]. AKT—protein kinase B; Bcl-2—B-cell lymphoma 2; BRAF—v-Raf murine sarcoma viral oncogene homolog B; cAMP—cyclic adenosine monophosphate; CDKN2A—cyclin-dependent kinase inhibitor 2A; CDKs—cyclin-dependent kinases; c-KIT—tyrosine-protein kinase KIT; c-MET—mesenchymal-epithelial transition factor; DAG—diacylglycerol; ERK—extracellular signal-regulated kinase; GF—growth factors; GPCR—G protein-coupled receptors; HIF—hypoxia-inducible factor; IGF-1—insulin-like growth factor 1; IKK—inhibitor of nuclear factor-κB (IκB) kinase; MAPK—mitogen-activated protein kinases; MC1R—melanocortin 1 receptor; MEK—mitogen-activated protein kinase; MITF—microphthalmia-associated transcription factor; mTOR—mammalian target of rapamycin; NF-1—neurofibromin 1; Nf-kB—nuclear factor kappa-light-chain enhancer of activated B cells; Notch—neurogenic locus notch homolog proteins; PI3K—phosphatidylinositol 3-kinase; PIP3—phosphatidylinositol 3-phosphate; PKC—protein kinase C; PLC—phospholipase C; PTEN—phosphatase and tensin homolog; RAS—“rat sarcoma virus” protein family; RTK—receptor tyrosine kinases; SCF—stem cell factor; SOX10—Sry-related HMg-Box gene 10; TNFR1—tumor necrosis factor receptor 1; WNT—“wingless-related integration site” protein family. Created with BioRender.com.

The pathogenesis of melanoma is believed to be closely related to the dysregulation of several molecular pathways caused by UV radiation (Figure 1). It is known that UV radiation causes the activation of various signaling pathways in skin cells [34]. This includes, among others: NRAS, phosphatidylinositol 3-kinase (PI3K), nuclear factor kappa-light-chain enhancer of activated B cells (NF-kB), microphthalmia-associated transcription factor (MITF), and the mitogen-activated protein kinases (MAPK) pathway [35,36,37]. It is especially true for MAPKs, which are usually overactivated in melanoma due to binding of growth factors to receptor tyrosine kinases (RTKs), as well as mutations of MAPK-related proteins that trigger a cascade of downstream molecular events [38,39].

Mutations in NRAS and BRAF genes, seen in a significant proportion of melanoma cases, lead to dysregulated signaling and persistent ERK activation, promoting cancer cell growth, melanoma development, and immune evasion [45]. BRAF mutations, particularly V600E, occur in approximately 40% of melanoma cases and 80% of melanocytic nevi, but alone are insufficient for disease initiation, indicating the potential involvement of additional genetic or environmental factors [42,46]. Similarly, NRAS mutations, especially the Q61R variant, appear in 10–25% of melanomas, affecting cell proliferation and survival [42]. The finding of an increased frequency of MAPK-related mutations and their biological consequences in various cancers, including melanoma, has led to the proposal of various MAPK suppression strategies, such as BRAF and MEK inhibition [47]. MAPK kinase inhibition with BRAF inhibitors such as vemurafenib has been shown to reduce melanoma cell proliferation and promote apoptosis, which has resulted in a clinically observable increase in progression-free survival (PFS) in melanoma patients [48]. However, the emergence of resistance and the increased risk of developing secondary skin cancers during BRAF inhibitor monotherapy have led to the inclusion of MEK inhibitors such as cobimetinib in the treatment regimens [48]. As a result, molecular therapy has not only prolonged the lives of patients with advanced melanoma but also improved their quality of life by reducing the adverse effects associated with conventional palliative chemotherapy [49,50]. However, despite the advances in targeted therapies using inhibitors of BRAF and MEK, therapeutic resistance may emerge because of various mechanisms, requiring combination therapies targeting multiple pathways [51]. In recent years, interventions at the genetic level, such as the prevention of alternative splicing of MAPK-related genes [52] or the blocking of genomic instability observed in the acquisition of resistance to therapy [53], have become an interesting area of research in the field of melanoma resistance to MAPK inhibition strategies. Furthermore, lipid metabolic reprogramming, which encompasses the inhibition of peroxisome biogenesis, modifications to ceramide metabolism [54], and an increase in the degree of unsaturation of membrane lipids [55], represents another promising avenue in the search for effective methods to overcome resistance mechanisms in melanoma cells. In this context, assessment of these interconnected pathways, their crosstalk, and their impact on gene regulation provides opportunities for further research to understand melanoma pathogenesis and develop effective treatment regimens.

A critical regulator in the MAPK pathway that plays a key role in melanocyte differentiation and melanoma malignancy is the microphthalmia-related transcription factor (MITF) [56], whose regulatory activities include genes integral to cell cycle progression, proliferation, and immune escape mechanisms [57]. Further complexity is added by genetic alterations within signal transduction genes—c-KIT, GNAQ/GNA11, and pigmentation-associated genes like MC1R, tyrosinase gene (TYR), tyrosinase-related protein 1 gene (TYRP1), and oculocutaneous albinism II gene (OCA2). Mutations in the aforementioned genes can drive melanoma development and progression by various mechanisms, including pigmentation deficiencies and hyper-activation of the downstream MAPK pathway [58,59].

In addition to the central role of the MAPK pathway, melanoma pathogenesis has also been found to depend on other pathways related to the above, such as PI3K-AKT and NFκB, and potentially linked to telomerase reverse transcriptase gene (TERT) expression [41]. Moreover, it is suggested that dysregulated signaling may result from overexpression or hyperactivation of other receptor tyrosine kinases (RTKs) such as c-Met, c-KIT, and EGFR [44,60]. It is believed that as a result of RAC-gamma serine/threonine-protein kinases (AKT1, AKT2, and AKT3) genes overexpression and loss of phosphatase and tensin homolog (PTEN), which play a key role in carcinogenesis and development of melanoma, the PI3K-AKT pathway is involved in the development of the majority of these cancers [61]. In a typical scenario, PI3 kinases phosphorylate PIP2 to PIP3, which leads to phosphorylation of protein kinase B (AKT) and activation of proteins that control cellular processes. However, loss or dysfunction of PTEN may even lead to constitutive activation of AKT, significantly contributing to the development of melanoma [62].

Another key pathway in melanoma pathogenesis is related to the cyclin-dependent kinase inhibitor 2A (CDKN2A). Mutations in CDKN2A promote uncontrolled cell-cycle progression, largely facilitated by the disruption of the retinoblastoma-associated protein (RB), cyclin D1 (CCND1), and cyclin-dependent kinase 4/6 (CDK4/6) [41]. Specifically, alterations of the CCND1 and CDK4/6 pathways have been identified as potential therapeutic targets, as they are more common in acral melanomas, subtypes associated with worse prognosis [63].

Moreover, the hypoxia-inducible factor (HIF) pathway, the Notch signaling pathway, the “wingless-related integration site” protein family (Wnt) signaling pathway, and NFκB activation contribute to the multifaceted network of dysregulated signaling in melanoma (Figure 1) [40]. These pathways regulate various cellular processes, including development, cell growth, migration, and differentiation, and their dysregulation has been linked to melanoma initiation, progression, and metastasis [64,65,66].

## 4. Development of UV-Dependent Melanoma

**Figure 2:**
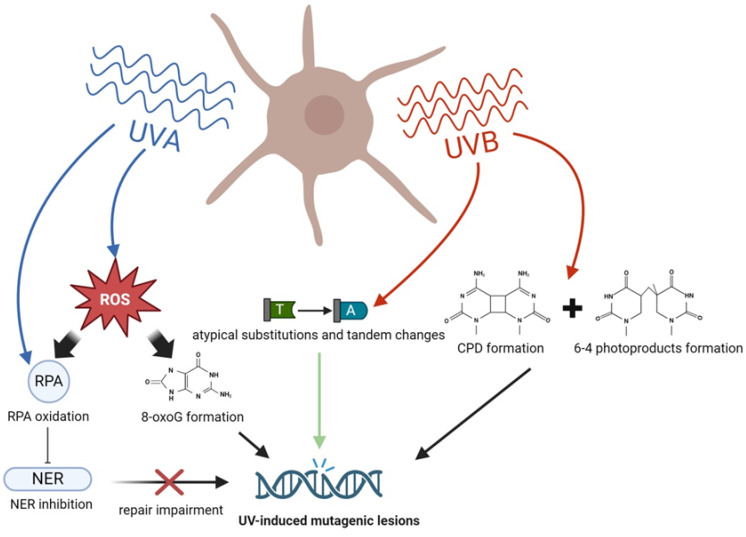
Mechanisms of the formation of UV-induced mutagenic lesions in melanocytes. ROS—reactive oxygen species, CPD—cyclobutane pyrimidine dimers, NER—nucleotide excision repair, T—thymine, A—adenine. Created with BioRender.com.

Ultraviolet radiation displays multifaceted influence on melanoma development due to its heterogenous nature, consisting of UVA (315–400 nm) and UVB (280–315 nm) radiation [67]. The different effects of each of the two UV ranges are based on their varying penetration depths and modes of DNA damage [68]. UVA, possessing longer wavelengths, penetrates more deeply into the skin, reaching the upper layers of dermis [69]. On the other hand, UVB, with its shorter wavelengths, directly damages DNA and is the main causative factor of melanoma [70]. UVB mainly promotes the formation of cyclobutane pyrimidine dimers (CPDs) and (6–4) photoproducts, especially at 5′TC and 5′CC DNA sites (Figure 2) [71]. These products occur when two adjacent pyrimidine bases in a DNA strand form covalent bonds with each other upon UV exposure, resulting in DNA distortion. Interestingly, a common UV signature found in melanoma genomes is the cytosine to thymine transition, primarily explained through two mechanisms: the error-prone replication model and the hydrolytic deamination model [72]. However, research indicates that the deamination of CPDs is the predominant factor behind most melanoma mutations [73]. Analogously, mutations in NRAS Q61R and BRAF V600E, which are seminal to melanomagenesis, do not correspond with the typical UV mutation signature, namely C > T transitions. Instead, these atypical mutations encompass substitutions such as T > A, T > C, and tandem changes like AC > TT, CT > TA, and CT > TC [35]. Such mutations, divergent from the UV-induced changes primarily involving CPD deamination, might originate from unique UV photoproducts (Figure 2) [35].

UVA, although less frequently, causes DNA damage either directly or indirectly via triplet energy transfer [74], typically occurring in TT sequences [75]. Furthermore, UVA irradiation leads to increased generation of reactive oxygen species (ROS), molecules responsible for cellular DNA, protein, and lipid modifications. A critical catalyst of this oxidative stress process triggered by UVA in melanocytes is pheomelanin. Upon exposure to UV radiation, pheomelanin undergoes photoexcitation, leading to enhanced ROS generation and DNA damages, such as 8-oxoguanine (8-oxoG) lesions, thereby playing a pivotal role in melanoma pathogenesis (Figure 2) [76,77].

The process of melanin degradation in response to UV exposure has also been shown to generate delayed high-energy products that transfer their energy to DNA, leading to the formation of “dark” cyclobutane pyrimidine dimers [78]. In contrast to “light” CPDs, the formation of such types of genetic lesions occurs hours after UV exposure [79]. This chemiexcitation process requires the presence of melanin and its fragments in the cell nucleus and could contribute to melanoma development [78]. Moreover, melanocytes with certain mutations like the inactive melanocortin 1 receptor (Mc1r) e/e allele are shown to produce more frequent CPD formations, suggesting a link between pheomelanin and a higher rate of DNA damage [78].

The impact of UV radiation on genetic material often results in dysfunction of exposed cells, even leading to their death and inflammation of neighboring tissue. Over time, accumulation of DNA alterations may constitute the basis for the melanoma development process [80]. Many melanoma cases display nucleotide excision repair (NER) system impairment, which is responsible for correcting those mutations in healthy melanocytes [81]. Disrupted NER function can result in conditions such as xeroderma pigmentosum, associated with an elevated risk of melanoma and other non-melanoma skin cancers [82]. NER operates through transcription-coupled repair (TCR), which rectifies transcription-blocking lesions, and global genome repair (GGR), responsible for repairing lesions throughout the genome. Notably, GGR plays a significant role in melanomagenesis, as certain NER-associated genes’ variations have been linked to the prognosis of melanoma patients [83].

Additionally, in the context of UV-induced DNA damage, the basal excision repair (BER) system cannot be omitted. BER typically repairs 8-oxoG lesions, which are commonly found in UVA-exposed melanocytes [84]. Loss of the BER-related 8-oxoguanine glycosylase (OGG1) gene enhances UVB-mediated carcinogenesis [85], highlighting the role of repair mechanisms in cancer prevention. Evidence that UVA radiation can damage DNA repair proteins through oxidation is exemplified by the potent oxidation of replication protein A (RPA), a crucial player in the ataxia telangiectasia and Rad3-related protein (ATR)-mediated pathway of NER activation (Figure 2) [86]. Moreover, melanocytes are found to have lower basal nucleotide excision repair activity than keratinocytes, which makes them exceptionally susceptible to photocarcinogenesis [87]. UV irradiation can not only influence melanoma development due to its mutagenic and repair-suppressive effects but could also affect melanoma cells fate and disease progression. This thesis is supported by the fact that in response to UVA radiation, melanoma cells show impaired NER, characterized by delayed expression of p53, DNA damage-binding protein 1 (DBB1), and DNA damage-binding protein 2 (DBB2) [88]. A visual summary of the broad UV irradiation effects on the melanocytic genome is presented in Figure 2. The green arrow represents atypical genetic alterations found in NRAS and BRAF mutations, likely arising from noncanonical UV photoproducts [35].

**Figure 3:**
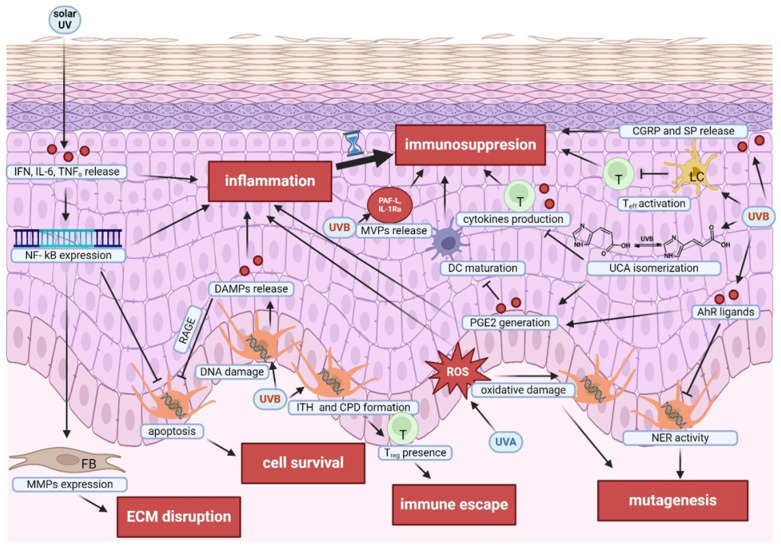
The effects of UV radiation leading to development of melanoma. FB—fibroblast. Created with BioRender.com.

The influence of UV radiation, especially UVB, on melanomagenesis is visible both at the level of metabolic changes in skin cells, especially keratinocytes, and in the modulation of the immune system [89]. It was found that after irradiation, keratinocytes react by releasing various mediators, creating an immunosuppressive environment that significantly hinders the functioning of both the innate and adaptive elements of the immune system (Figure 3) [90].

One of such immunosuppressive mechanisms induced by UVB involves the isomerization of urocanic acid (UCA), leading to increased generation of prostaglandin E2 (PGE2), which combined promote the formation of an immunosuppressive environment [91]. UV radiation also impairs the antigen-presenting function of Langerhans cells (LCs) [92], thus reducing the stimulation of effector T cells, a critical aspect of the adaptive immune response [93]. Additionally, UVB radiation stimulates the production of tryptophan photoproducts, mainly 6-formylindolo [3,2-b]carbazole (FICZ)—the agonist of the aryl hydrocarbon receptor (AhR) [94]. However, AhR activation results in increased cyclooxygenase-2 (COX-2) expression, further exacerbating the immunosuppressive effect of UV irradiation [95]. In addition, AhR influences melanomagenesis by inhibiting the NER system, leading to a subsequent increase in DNA structure alterations (Figure 3) [96].

UV radiation not only induces damage at the nucleic acid level but also impacts the quantity and biological effectiveness of proteins. One of the mechanisms of this phenomenon includes the excessive generation of reactive oxygen and reactive nitrogen species (ROS/RNS) due to endogenous chromophores photosensitization [97]. ROS suppress the immune response in the tumor microenvironment (TME) and are strongly associated with inflammation and oxidative stress (Figure 3) [98]. The inflammatory process involves the activation of specific pathways, such as NF-kB and p38MAPK, which are active in various cell types, both immune and non-immune cells [99]. It has been shown that sunburn, a common reaction to excessive UV exposure, can activate the Toll-like receptor (TLR) cascade, leading to increased production of immune mediators such as interferon (IFN), interleukin 6 (IL-6), and tumor necrosis factor α (TNFα) [100,101]. Increased TNFα level caused by acute exposure to UV irradiation promotes intense activation of the NF-kB transcription factor [102], responsible for pro-inflammatory and anti-apoptotic signaling in melanoma development [103]. Melanocytes also participate in radiation-induced inflammation by releasing damage-associated molecular proteins (DAMPs), which are part of the DNA damage response and activate the innate immune response [104]. In addition, DAMPs interact with the receptor for advanced glycosylation end products (RAGE), and its activation contributes to melanocyte resistance to apoptosis, potentially promoting melanoma initiation (Figure 3) [104].

However, in the case of chronic UVB exposure, prolonged inflammative response can stimulate the immunosuppressive regulatory T lymphocytes (Tregs) and myeloid-derived suppressor cells (MDSCs), impacting melanoma progression [105,106]. Furthermore, UVB radiation stimulates the formation of microvesicle particles (MVPs) from the keratinocyte plasma membrane [90]. These MVPs transport bioactive substances, such as platelet-activating factor (PAF)-like ligands and interleukin-1 receptor antagonist IL-1Ra, that can contribute to systemic immunosuppression after UV exposure (Figure 3) [90]. Moreover, UV-enhanced NF-kB signaling increases extracellular matrix (ECM) degradation by upregulating metalloproteinase (MMP) activity [107]. ECM disruption, including collagen fragmentation and lowered levels of collagen cross-linking proteins, can facilitate tumor invasion and metastasis [108]. The immunosuppressive environment generated by UV radiation significantly hampers the immune system’s capacity to detect and respond to abnormal cells, including potentially cancerous cells. This effect is especially significant in immunocompromised patients who are at higher risk for developing melanoma and other skin cancers [109]. Additionally, UVB exposure enhances melanoma tumorigenicity through inducement of intratumor heterogeneity (ITH) [110]. ITH can affect immune surveillance, where higher ITH is associated with an increased presence of Tregs in the tumor microenvironment [111], potentially impacting the efficacy of immunotherapies such as immune checkpoint inhibitors [110]. Notably, melanomas in skin areas with high sun exposure have more UVB signature mutations and a higher tumor mutational burden (TMB), factors that increase immunogenicity and improve responses to anti-PD1 immunotherapy [112]. Another mechanism by which UVB radiation influences the immune system by inflicting DNA damage is through the generation of CPDs, which trigger a strong immunosuppressive response, possibly contributing to skin carcinogenesis [113].

UVB radiation can stimulate the generation of various immune-regulatory molecules like interleukins, chemokines, and COX-2 [114]. These molecules play a crucial role in the immune response to UV radiation. For instance, prostaglandin E2 (PGE2), a potent inflammatory mediator, regulates inflammation and activates immune cells, yet in some cases, it also inhibits the maturation of dendritic cells and their ability to activate T cells (Figure 3) [115]. Interestingly, interleukin 12 (IL-12), a potent immune-stimulating cytokine, can potentially mitigate UV-induced immunosuppression by counteracting the effects of DNA damage [116]. This notion is supported by experimental studies showing that IL-12 injection can successfully treat melanoma in mouse models [117]. Additionally, UV radiation can stimulate keratinocytes to secrete neuropeptides, such as calcitonin gene-related peptide (CGRP) and substance P (SP) [118]. These neuropeptides further activate mast cells responsible for regulating antigen presentation, thereby affecting the overall immune response [119]. The multifaceted role of UV irradiation in melanoma development is summarized in Figure 3.

## 5. Redox Status in the Initiation and Subsequent Development of Melanoma

The redox status plays a crucial role in the initiation and subsequent development of melanoma. Elevated levels of reactive oxygen species (ROS) can induce oxidative stress, leading to DNA damage and mutations that drive the transformation of melanocytes into malignant melanoma cells. The altered redox balance influences key signaling pathways, promoting cell proliferation, survival, and metastatic potential. Moreover, the redox environment modulates the tumor microenvironment, enhancing angiogenesis and immune evasion. Understanding the dynamics of redox status in melanoma can reveal novel therapeutic targets and strategies for effective intervention.

**Figure 4:**
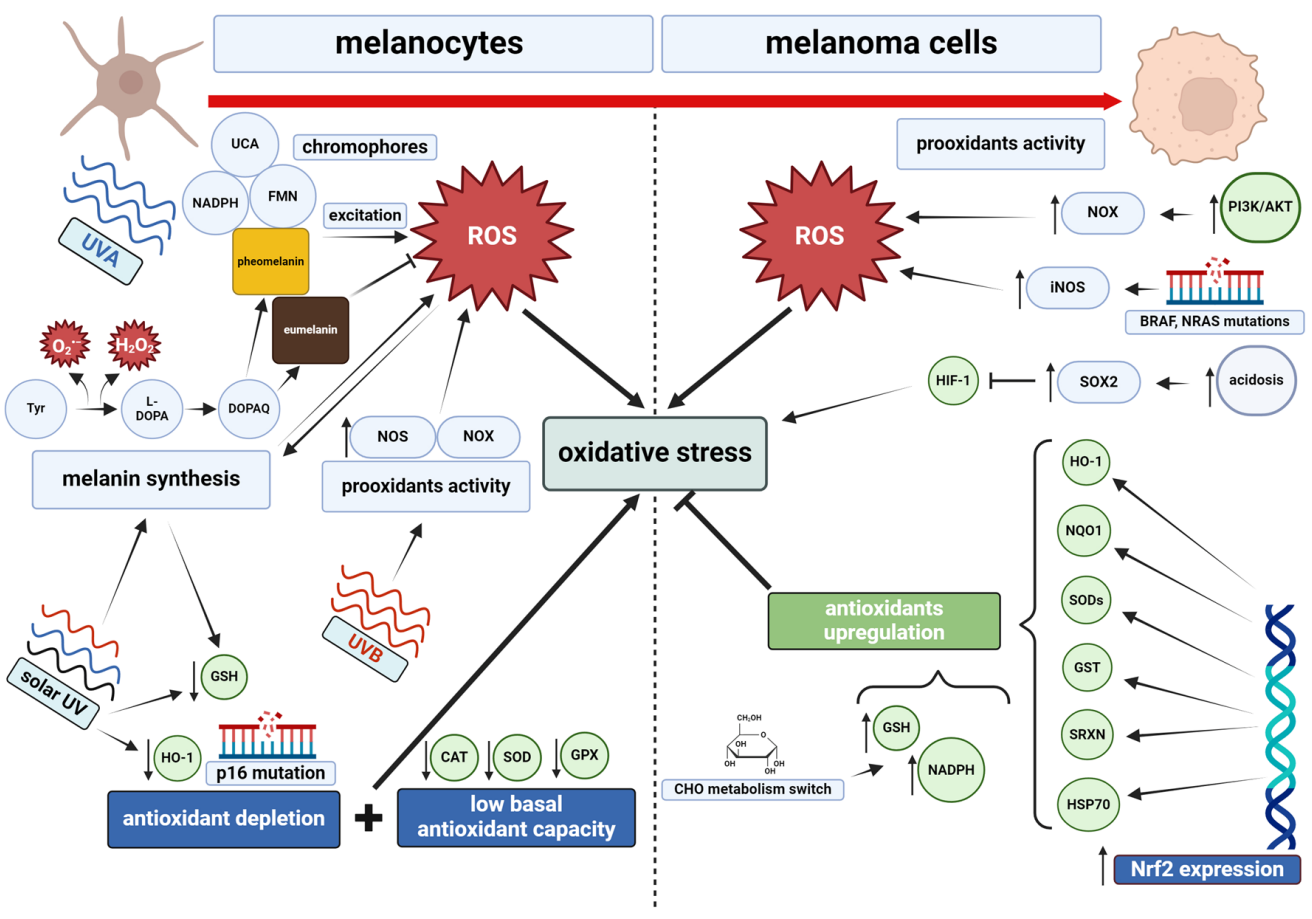
Differences in redox status over the course of melanomagenesis. CHO—carbohydrates, DOPAQ—dopachinone, FMN—flavin mononucleotide, HIF-1—hypoxia-induced factor 1, Tyr—tyrosine. Created with BioRender.com.

One of the primary causes of skin cancer development, including melanoma, are metabolic disturbances within skin cells, especially in redox equilibrium. In the case of melanocytes and melanoma cells, oxidative stress mainly results from the influence of solar UV radiation on cellular metabolism (Figure 4).

### 5.1. The Impact of Melanin and Melanogenesis on the Redox Homeostasis of Melanocytes

Melanocytes, precursor cells of melanoma, are prone to overproduction of ROS due to the continuous biosynthesis of melanin and stimulation of endogenous chromophores (e.g., flavins, melanins) in response to solar UVA radiation [120,121]. Biosynthesis of melanin relies on enzymatic oxidation of tyrosine to l-3,4-dihydroxyphenylalanine (L-DOPA), a process that generates ROS (namely superoxide anion and hydrogen peroxide) and further exacerbates prooxidative melanocytic environment [122]. Moreover, UVA stimulates pigment synthesis through upregulation of mitochondrial ROS [123], suggesting the presence of mutual dependencies between the mechanisms leading to the disruption of redox balance in melanocytes.

It has been shown that melanocytes with higher levels of pheomelanin compared with eumelanin, resulting in lighter skin pigmentation, are more susceptible to the effects of ROS and consequently, have an increased risk of carcinogenesis [124]. The synthesis of pheomelanin, compared with eumelanin, promotes the generation of a larger quantity of ROS, and the pigment itself exhibits heightened prooxidative properties after sunlight exposure [125]. UVB radiation, abundant in solar light, induces expression of inducible nitric oxide synthase (iNOS) and NADPH oxidase 1 (NOX1) [126], prooxidant proteins tightly related to melanomagenesis. Increased activity of NOS and NOX is specific to pigmented melanocytes, in which their action can lead to a shift in melanin synthesis from eumelanogenesis to pheomelanogenesis, contributing to the development of melanoma from dysplastic nevus [127]. Dysplastic nevi, characterized by increased pheomelanin synthesis [128], are well-recognized melanoma risk factors [129]. These discrepancies are exacerbated in melanocytes with lower levels of eumelanin, characterized by reduced antioxidant activity compared with melanocytes with higher levels of this pigment [130].

Melanin is also capable of forming complexes with xenobiotics, including commonly used antibiotics such as fluoroquinolones and tetracyclines [131]. These drugs, when combined with UVA irradiation, have been observed to exert phototoxic effects on melanocytes, resulting in the generation of oxidative stress [132,133]. In the case of chlortetracycline, there is evidence that it additionally increases tyrosinase activity, which in turn stimulates melanogenesis [132]. Therefore, it can be suggested that there is an elevated risk of melanoma development during therapy with the aforementioned drugs, as evidenced by epidemiological studies on melanoma incidence in fluoroquinolone users [131], highlighting the importance of melanin contribution to melanomagenesis.

### 5.2. The Contribution of Oxidative Stress to the Initiation of Melanoma

In comparison to other normal skin cells, melanocytes exhibit heightened susceptibility to the ionizing effects of UVA radiation, manifested by elevated production of 8-oxoG and overexpression of BER [134]. Furthermore, pigmented cells are more vulnerable to the action of oxidants, owing to reduced activity of canonical antioxidant systems resulting from the action of UVA and UVB radiation [135]. This phenomenon is associated with reduced biosynthesis and activity of antioxidant proteins, including catalase (CAT), superoxide dismutase (SOD), and glutathione peroxidase (GPX), which all display reduced activity in irradiated melanocytes [130]. Daily exposure to sunlight not only upregulates melanogenesis, which contributes to increased depletion of cysteine and reduced glutathione (GSH) as a result of their reaction with dopaquinone (DOPAQ), a precursor of pigments [136], but also downregulates the expression of heme oxygenase 1 (HO-1) [130]. Therefore, compromised antioxidant mechanisms, coupled with a heightened tendency to generate ROS, disrupt the redox balance in melanocytes (Figure 4). Further support for this claim is the fact that melanocytes are more susceptible to CDKN2A deficiency than keratinocytes or fibroblasts [43]. Reduced activity of the p16 protein, typically involved in inhibition of ROS generation, may significantly intensify oxidative stress, potentially explaining the link between the INK4A mutation and melanoma development [137].

In contrast, UV-induced oxidative stress serves as a key initiator of melanocyte apoptosis, a crucial mechanism underlying the pathogenesis of vitiligo, an autoimmune depigmentary disease [138]. Studies have demonstrated that the excessive generation of reactive oxygen species (ROS) due to UV irradiation and chronic inflammation results in an imbalance in the antioxidant defense system, leading to cell damage and death in vitiligo patients’ melanocytes [139,140]. It can be hypothesized that, in contrast to the pathogenesis of vitiligo, a specific basal level of antioxidant activity is crucial in the melanoma initiation. This ensures the survival of melanocytes in an oxidative stress environment, yet does not protect them from non-lethal oxidative modifications of nucleic acids, proteins, and lipids, which accumulate and ultimately lead to neoplastic transformation.

### 5.3. The Impact of Oxidative Stress on Melanomagenesis

It is known that prolonged UV irradiation of melanocytes results in a shift in redox balance towards pro-oxidant conditions, which promotes the proliferation of pre-cancerous melanocytes [141]. Furthermore, DNA hypermethylation triggered by oxidative stress inhibits anoikis, a form of apoptosis that occurs when melanocytes detach from the extracellular matrix, which is a key step in melanoma development [142]. However, increased expression of sestrin 2 (Sesn2) and the p65 subunit of NF-kB leads to excessive activation of the AKT pathway, thus inhibiting the apoptosis of pre-cancerous melanocytes and intensifying the modification and accumulation of changed cells (Figure 4) [143,144].

**Figure 5:**
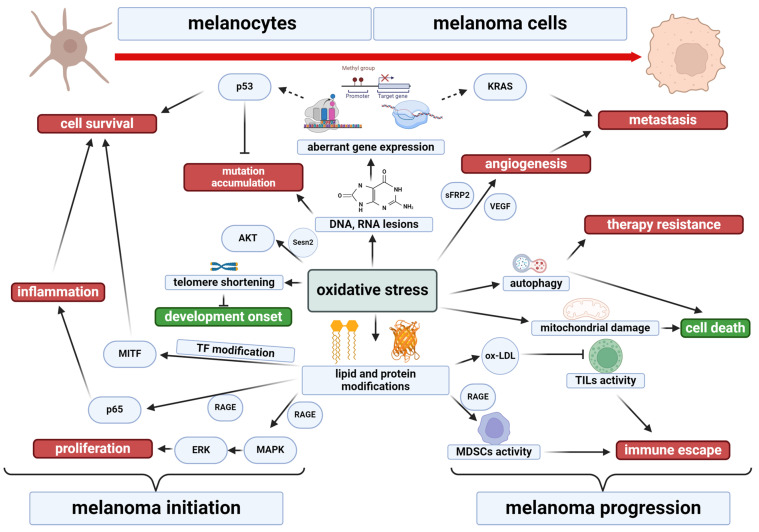
The impact of oxidative stress on melanomagenesis. TF—transcription factor. Created with BioRender.com.

However, in the case of melanoma cells, the exact relationship between redox status and disease progression is not clear (Figure 5). Oxidative stress, characterized by a shift in the redox balance toward oxidative reactions, significantly affects melanomagenesis, acting as a double-edged sword. High levels of ROS can contribute to the increased aggressiveness of various cancer cells, promoting their ability to proliferate, invade, and migrate [145]. ROS are known to promote angiogenesis and tumor metastasis in melanoma by increasing the expression of secreted frizzled-related protein 2 (sFRP2) and vascular endothelial growth factor (VEGF) [146,147]. In addition, elevated levels of ROS affect macrophage activity, which promotes tumor invasion [148]. The NADPH oxidase (NOX4) acts as a survival signal for melanoma cells, maintaining adhesion contacts and cell viability through the focal adhesion kinase (FAK) pathway (Figure 5) [149].

As a result of oxidative stress, the functioning of cells at the molecular level is impaired, which is related to the oxidation of key organic compounds—nucleic acids, proteins, and lipids. DNA, the double-stranded nucleic acid that determines the amino acid sequences of cellular proteins, is particularly sensitive to oxidative damage. 8-oxoG is the primary oxidative lesion found in DNA structure [150] responsible for the occurrence of substitution mutations and strand cross-linking in the presence of deficient repair systems, resulting in altered transcription and translation [151,152]. Importantly, changes observed in the aforementioned processes can significantly promote the initiation and progression of malignancies, including gastric and breast cancer [153,154]. Furthermore, 8-oxoG also regulates gene expression at the epigenetic level [155], promoting transcription of KRAS in pancreatic cancer cells [156], which is an oncogene associated with brain metastasis risk in melanoma patients (Figure 5) [157]. Another nucleic acid, RNA, is also susceptible to oxidative 8-oxoG modifications, leading to disruption of gene expression even at the ribosomal level [158]. RNA alterations observed in human bronchial epithelial cells under oxidative conditions reduced the expression of p53 [159], a tumor suppressor whose inactivation is a key step in melanomagenesis [160].

DNA damage resulting from the oxidative stress environment also includes shortening of telomeres, the repetitive nucleotide sequences that protect the ends of strands from excessive degradation [161]. Regarding melanoma, telomere length correlates positivelywith patient survival [162]; however, telomere dysfunction and associated genome destabilization delay tumor development in BRAF V600E mutated mice [163].

At the same time, high levels of ROS can induce apoptosis and senescence of cancer cells through oxidative damage to DNA and mitochondrial pathways, suggesting their tumor suppressive role [41,164]. Underlying these effects is the activation of different cellular pathways. For example, a common BRAF mutation observed in melanoma (V600E) regulates oxidative metabolism through peroxisome proliferator-activated receptor–gamma coactivator 1-α (PGC1α) and MITF, linking increased mitochondrial capacity and resistance to oxidative stress [165]. In addition, ROS modulate oxidative stress signaling pathways such as MAPK, PI3K/AKT, and NF-κB [166,167] and nuclear factor erythroid 2-related factor 2 (Nrf2)-dependent antioxidant pathways [168]. Alterations at the genomic level, such as PTEN and MC1R mutations, further exacerbate oxidative stress in melanoma [141,169]. Interactions between oxidative stress and the process of autophagy, in which oxidized forms of various compounds are degraded, further regulate the negative consequences of oxidative stress, showing complex relationships that, in combination with genetic mutations such as BRAFV600E and PTEN-null, can modulate the fate of cancer cells (Figure 5) [170]. This relationship is being intensively studied to find new strategies for melanoma therapy using experimental drugs and photobiomodulation [171,172]. Furthermore, the effects of oxidative stress extend to the regulation of miRNA profiles, non-coding RNAs involved in the regulation of gene expression [173], suggesting promising therapeutic targets for melanoma treatment.

Multifaceted effects exerted by oxidative stress on proteins include oxidation of amino acids’ thiol groups, carbonylation, and glycosylation [174]. Although glycosylation occurs slowly under physiological conditions, the oxidative stress environment of UV-irradiated skin accelerates the formation of advanced glycation end products (AGEs) and promotes their accumulation in the dermis (Figure 5) [175]. AGEs act through receptor for advanced glycation end products (RAGE) to further trigger the inflammatory response, leading to activation of melanocyte MAPK signaling [176], which is implicated in melanoma initiation. The AGEs–RAGE axis is also responsible for regulating the immune response against melanoma by promoting MDSC suppressing activity, consequently increasing the number of metastases and mortality in the mouse model [177]. Moreover, ROS-mediated cysteine oxdiation in MITF transcription factor leads to enhanced expression of its downstream effector genes, which contribute to cancerous transformation in MC1R defective melanocytes (Figure 5) [178].

### 5.4. The State of Redox Balance in Melanoma Cells

Literature data indicates that the shift of the redox balance towards oxidative reactions accompanying the neoplastic transformation of melanocytes also extends to modifications of metabolic processes in melanoma cells. The progression of melanoma is intrinsically linked to changes in cellular biology that determine the invasive phenotype of malignant cells. This is characterized by altered expression of membrane proteins (e.g., cadherins, immunogenic antigens), which conditions melanoma cells’ ability to migrate, metastasize, and evade the host immune response. These processes are crucial for further disease progression and are substantially modified by an oxidative stress environment.

ROS generation in melanoma cells is intensified by various factors, including the stimulation of PI3K/AKT signaling pathways [179], hypoxia [180], and altered metabolic processes within mutated cells, with the central role of NOS and NOX prooxidant activity as general elements of melanoma progression [181]. Upregulation of NOX5 expression enhances cell proliferation and resistance to BRAF inhibition therapy [182], while NOX1 overexpression favors melanoma invasion [183]. NOX4, highly expressed in metastatic sites, contributes to the transformation of cellular phenotype and affects melanoma cell cycle progression [184]. Additionally, NOS intensifies oxidative stress, influencing melanoma progression through amplified cell proliferation, invasiveness, and chemotherapy resistance [185]. Furthermore, certain mutations within BRAF and NRAS melanoma-associated genes lead to overexpression of iNOS, subsequently promoting malignant cell survival through NO release and anti-apoptotic signaling (Figure 4) [127].

To counteract the negative effects of excessive ROS generation, melanoma cells display intensified activity of several components of the antioxidant system. Transcription factor Nrf2 plays a central role in maintenance and regulation of redox equilibrium (Figure 4). Nrf2 coordinates the antioxidant response, working as a master regulator of genes related to cytoprotection against oxidative damage. Its essential influence on melanomagenesis is reflected by regulation of tumor invasion depth and patient survival prognostics [186]. Downstream Nrf2 signaling regulates MITF activity and EGFR expression, thus highlighting the crosstalk between intracellular communication and oxidative stress management in melanoma [187]. It has been observed that the main products of Nrf2 transcriptional activity, heme oxygenase-1 (HO-1), as well as NAD(P)H:quinone oxidoreductase 1 (NQO1) [188], can undergo overexpression in melanoma [189], influencing its development by increasing cell proliferation and resistance to oxidative stress and therapy [190,191]. Moreover, there are several other components of Nrf2-dependent antioxidant systems that participate in melanomagenesis through counteracting melanoma ROS overproduction. These include superoxide dismutases (cytosolic SOD1 and mitochondrial SOD2), which convert superoxide anion into hydrogen peroxide and are upregulated by Nrf2 in melanoma cells [189,192]. Additionally, Nrf2-related enzymes such as glutamate-cysteine ligase (GCL) and glutathione synthetase (GHS) participate in the biosynthesis of the key antioxidant peptide, glutathione [193]. Glutathione provides electrons for glutathione peroxidase (GPx) to reduce hydrogen peroxide and lipid peroxides, while glutathione S-transferases (GSTs) detoxify electrophilic molecules through conjugation with glutathione [194]. It is worth emphasizing that melanoma cells often exhibit elevated GST activity, contributing to cell proliferation and the formation of metastases [195]. Thioredoxin (TRX) and thioredoxin reductase (TrxR), which form a system that reduces oxidatively modified cysteine, also remain under the transcriptional control of Nrf2 [196]. Downregulation of TRX inhibitor-thioredoxin-interacting protein (TXNIP) promotes melanoma progression and lung metastases [197], while TRX expression and secretion to TME generate immunotolerance [198]. Moreover, TrxR is overexpressed in melanoma cells, and targeting its function could have implications in melanoma therapy [199]. Another group of Nrf2-dependent antioxidant enzymes, sulfiredoxins (SRXNs), regulate oxidative stress in cells by assisting peroxiredoxins (PRDXs) in controlling hydrogen peroxide levels [200], are overexpressed in melanoma cells and participate in the malignancy’s initiation [201]. Heat shock protein 70 (HSP70) is also involved in attenuating the negative effects of oxidative stress, and its expression can also be regulated by Nrf2 [202]. In melanomas, HSP70 protects cells from apoptosis and positively regulates BRAF pathways, creating favorable conditions for further melanoma development [203].

The Nrf2 factor remains in metabolic cooperation with nuclear factor kappa B (NF-κB), a transcription factor involved in the immune response, inflammation, and progression of malignancies, including melanoma (Figure 4) [204]. In melanoma cells, mutations in the BRAF gene and anomalies in signaling pathways involving PI3K, AKT, and RAS favor the constitutive activity of NF-κB, resulting in enhanced proliferation and apoptosis resistance [205]. In addition, clinically elevated NF-κB levels were found to correlate with increased angiogenesis in melanoma tumors, highlighting its importance in disease progression [206]. An imbalance between Nrf2- and NFkB-regulated pathways can result in inflammation and disease [207] due to NF-κB-mediated suppression of anti-inflammatory genes and overexpression of Nrf2 affecting NF-κB activation [208]. The p65 protein, a REL-related protein crucial for NF-κB activity, has been shown to simultaneously antagonize Nrf2 function with some compounds, for example cannabidiol, suppressing p65 while enhancing the Nrf2 pathway [209]. Activation of Nrf2 inhibits the production of inflammatory molecules [210] and increases intracellular glutathione levels [211], thereby reducing oxidative conditions and inhibiting the action of NF-κB.

In addition to the regulation of antioxidant activities related to the efficiency of the transcription factor Nrf2, melanoma cells also modify carbohydrate metabolism to counteract increased ROS production (Figure 4). This mainly involves upregulation of the pentose-phosphate pathway and serine biosynthesis, which play a significant role in the production of NADPH and GSH [212,213]. Melanomas with mutated glucose-6-phosphate dehydrogenase (G6PD), a key enzyme in the pentose phosphate pathway, were found to have higher levels of oxidative stress, highlighting G6PD inhibition as a potential therapeutic target [212].

In addition to changes at the genome and proteome levels, oxidative stress in melanoma cells promotes ROS interactions with cellular lipids, particularly their polyunsaturated fatty acids (PUFAs), in the process known as lipid peroxidation [214]. One of its products, 4-hydroxynonenal (4-HNE), is noticeably elevated in dysplastic nevi and melanomas compared with benign nevi [215], potentially influencing melanomagenesis through the formation of DNA adducts (Figure 5) [216]. Lipid peroxidation also contributes to the evasion of the immune response by melanoma cells due to the pro-oxidative conditions in the TME [217]. CD8+ tumor-infiltrating lymphocytes (TILs) uptake the oxidized LDL (ox-LDL) found in the TME, leading to reduced production of pro-inflammatory cytokines, which supports further melanoma progression [217]. Moreover, oxidative stress influences PUFAs enzymatic metabolism through upregulation of peroxiredoxin 6 (PRDX6) expression [218]. PRDX6 displays calcium-independent phospholipase A2 (iPLA_2_) activity, which increases melanoma cell proliferation through arachidonic acid (AA) release and its downstream signaling [219]. Noteworthy, cyclooxygenases (COX) and lipoxygenases (LOX), enzymes responsible for the conversion of AA to eicosanoids, are constitutively expressed in melanoma lesions, and their selective inhibition reduces cancerous cell viability, with 5-LOX inhibitors also exerting antiproliferative effects [220]. However, UVA- and UVB-induced oxidative stress in skin fibroblasts decreases cellular levels of endocannabinoids, another PUFA-derived lipid mediator found also in melanoma cells [221,222]. The exact role of endocannabinoids in melanomagenesis is not yet fully elucidated, but they can diversely affect cellular redox balance based on activation of pro-oxidant cannabinoid receptor 1 (CB1) or antioxidant cannabinoid receptor 2 (CB2) [223]. Furthermore, modulation of the endocannabinoid system was found to inhibit melanoma metastases by inhibition of cell proliferation and angiogenesis [224]. Therefore, studies regarding the contribution of eicosanoids and endocannabinoids in melanomagenesis, especially in the context of redox balance disturbances found in precancerous melanocytes and melanoma cells, hold promise for future improvements in diagnosis and therapy of the disease [225].

## 6. Conclusions

In conclusion, melanomagenesis is a multistep, heterogenous process driven by the neoplastic transformation of melanocytes, which is primarily dependent on UV radiation. This statement is based on a metabolic analysis of the biological effects of UV irradiation, including the overproduction of ROS, DNA damage, immunosuppression, and degradation of ECM. When considered alongside epidemiological evidence, these factors allow us to conclude that UV radiation is the most significant factor in the development of melanoma. Therefore, this study presents a comprehensive review of the impact of UV-induced metabolic alterations, specifically resulting oxidative stress in melanocytes, which serves as the foundation of their neoplastic transformation and an important modulator of further disease progression. However, the lack of clear data and the existence of conflicting literature reports regarding the direct correlation between the oxidative modification of melanocyte macromolecules and the subsequent carcinogenesis indicate that the presented findings are not yet conclusive. Instead, it represents an overview of available data and suggestions for further research, which may facilitate the establishment of a precise connection between oxidative modifications and melanomagenesis in the near future.

Currently, it can be clearly stated that oxidative stress, resulting from the UV exposure of the skin, including melanocytes, vastly participates in the initiation of the melanomagenesis, which indicates the possibility of a rational use of antioxidants for the prevention of melanoma. However, the exact role of oxidative stress in the context of further disease progression remains unclear, as melanoma cells display a delicate balance between elevated ROS and RNS levels and upregulation of enzymatic antioxidants. On the one hand, elevated expression of pro-oxidant proteins facilitates melanoma cell invasion and metastasis and correlates positively with increased angiogenesis or resistance to specific therapeutic agents. Conversely, the upregulation of Nrf2-related antioxidant systems, the shift of energy metabolism toward antioxidant regeneration, and the reduction of oxidative phosphorylation rates in favor of glycolysis are thought to indicate a multifaceted effort made by melanoma cells to reduce oxidative stress. Thus, an analysis of the redox balance during melanomagenesis suggests that an oxidative stress environment may favor the neoplastic transformation of melanocytes while simultaneously creating undesirable conditions for the subsequent advancement of the disease. The positive correlations observed between the expression of genes responsible for protection against oxidative damage and the proliferation, invasiveness, and resistance to therapy of melanoma cells emphasize the importance of research on regulating the redox balance in melanoma progression.

Consequently, it can be suggested that the metabolic adaptations observed throughout the melanomagenesis constitute the basis for attempts to utilize redox balance modulators, such as prooxidants, in melanoma therapy and in strategies to overcome treatment resistance. Therefore, further research into the pathophysiological basis of melanoma development gives hope for the discovery of potential new target factors and the identification of compounds capable of adjusting the related metabolic changes in order to promote melanoma cell apoptosis driven by oxidative stress. However, a better understanding of these multidirectional biological mechanisms may support the development of new therapeutic strategies and enhance the effectiveness of current treatment regimens, which will ultimately lead to increased patient survival.

## Figures and Tables

**Table 1:** Types of melanoma caused by exposure to UV radiation.

Melanoma Type	UV Exposure	Anatomical Site	Average Age at Diagnosis	Predominant Mutations	References
High-CSD	Prolonged	Head, neck, distal extremities	>55 years	NRAS, KIT, NF-1, ROS-1, BRAF, GNA 11, RAC1 mutations	[17,23]
Low-CSD	Intermittent	Trunk, proximal extremities	<55 years	BRAF_V600E_, RAS mutations	[17,23]
Non-solar	Not significant	Mucous membranes, uvea, nevi	Varies (from average 15 years in congenital nevi malignancy to 70 years in mucosal melanoma)	Varies (UM - GNAQ, GNA11 mutations MM - KIT, SF3B1 mutations NAM - BRAF, NRAS mutations)	[24,25,26,27]

GNAQ- Guanine nucleotide-binding protein G(q) subunit alpha; GNA11- Guanine nucleotide-binding protein subunit alpha-11; RAC1- Ras-related C3 botulinum toxin substrate; SF3B1- splicing factor 3B subunit 1; UM—uveal melanoma; MM—mucosal melanoma; NAM—nevi-associated melanoma.

## Abbreviations

4-HNE	4-hydroxynonenal
5-LOX	5-lipooxygenases
8-oxoG	8-oxoguanine
A	adenine
AA	arachidonic acid
AGE	advanced glycation end products
AKT	protein kinase B
ALM	acral lentiginous melanoma
AhR	aryl hydrocarbon receptor
BER	basal excision repair
BRAF	v-Raf murine sarcoma viral oncogene homolog B
Bcl-2	B-cell lymphoma 2
C	cytosine
CAT	catalase
CB1	cannabinoid receptor 1
CB2	cannabinoid receptor 2
CCND1	cyclin D1
CDK 4/6	cyclin-dependent kinase 4/6
CDKN2A	cyclin-dependent kinase inhibitor 2A
CDKs	cyclin-dependent kinases
CGRP	calcitonin gene-related peptide
CHO	carbohydrates
COX	cyclooxygenases
COX-2	cyclooxygenase-2
CPD	cyclobutane pyrimidine dimers
CSD	chronic sun damage
DAG	diacylglycerol
DAMPs	damage-associated molecular proteins
DBB1	damage-specific DNA binding protein 1
DBB2	damage-specific DNA binding protein 2
DM	desmoplastic melanoma
DOPAQ	dopachinone
ECM	extracellular matrix
EGFR	epidermal growth factor receptor
ERK	extracellular signal-regulated kinase
FAK	focal adhesion kinase
FICZ	6-formylindolo [3,2-b]carbazole
FMN	flavin mononucleotide
G6PD	glucose-6-phosphate dehydrogenase
GCL	glutamate-cysteine ligase
GF	growth factors
GGR	global genome repair
GHS	glutathione synthetase
GNA11	guanine nucleotide-binding protein subunit alpha-11
GNAQ	guanine nucleotide-binding protein G(q) subunit alpha
GPCR	G protein-coupled receptors
GPX	glutathione peroxidase
GSH	glutathione
GSTs	glutathione S-transferases
HIF	hypoxia-inducible factor
HIF-1	hypoxia-induced factor 1
HO-1	heme oxygenase 1
HSP70	heat shock protein 70
IFN	interferon
IGF-1	insulin-like growth factor 1
IKK	inhibitor of nuclear factor-κB (IκB) kinase
IL-12	interleukin 12
IL-1Ra	interleukin-1 receptor antagonist
IL-6	interleukin 6
ITH	intratumor heterogeneity
L-DOPA	l-3,4-dihydroxyphenylalanine
LCs	Langerhans cells
LMM	lentigo maligna melanoma
LOX	lipooxygenases
MAPK	mitogen-activated protein kinases
MC1R	melanocortin 1 receptor
MDSCs	myeloid-derived suppressor cells
MEK	mitogen-activated protein kinase
MITF	microphthalmia-associated transcription factor
MM	mucosal melanoma
MMPs	metalloproteinases
MVPs	microvesicle particles
NAM	nevi-associated melanoma
NER	nucleotide excision repair
NF-1	neurofibromin 1
NM	nodular melanoma
NOX1	NADPH oxidase 1
NOX4	NADPH oxidase 4
NOX5	NADPH oxidase 5
NQO1	NAD(P)H:quinone oxidoreductase 1
Nf-kB	nuclear factor kappa-light-chain enhancer of activated B cells
Notch	neurogenic locus notch homolog proteins
Nrf2	nuclear factor erythroid 2-related factor 2
OCA2	oculocutaneous albinism II gene
OGG1	8-oxoguanine DNA glycosylase-1
PAF-L	platelet-activating factor ligands
PGC1α	peroxisome proliferator-activated receptor-gamma coactivator 1-α
PGE2	prostaglandin E2
PI3K	phosphatidylinositol 3-kinase
PIP3	phosphatidylinositol 3-phosphate
PKC	protein kinase C
PLC	phospholipase C
PRDX	peroxiredoxins
PRDX6	peroxiredoxin 6
PTEN	phosphatase and tensin homolog
PUFAs	polyunsaturated fatty acids
RAGE	receptor for advanced glycosylation end products
RAS	“rat sarcoma virus” protein family
RB	retinoblastoma-associated protein
REL	proto-oncogene c-Rel
RNS	reactive nitrogen species
ROS	reactive oxygen species
RPA	replication protein A
RTK	receptor tyrosine kinases
SCF	stem cell factor
SOD	superoxide dismutase
SOX10	Sry-related HMg-Box gene 10
SP	substance P
SRXN	sulfiredoxins
SSM	superficial spreading melanoma
Sesn2	sestrin 2
T	thymine
TCR	transcription-coupled repair
TILs	tumor-infiltrating lymphocytes
TLR	Toll-like receptor
TMB	tumor mutational burden
TME	tumor microenvironment
TNFR1	tumor necrosis factor receptor 1
TNFα	tumor necrosis factor α
TRX	thioredoxin
TXNIP	thioredoxin-interacting protein
TYR	tyrosinase
TYRP1	tyrosinase-related protein 1
Tregs	regulatory T lymphocytes
TrxR	thioredoxin reductase
Tyr	tyrosine
UCA	urocanic acid
UM	uveal melanoma
UVA	ultraviolet radiation A
UVB	ultraviolet radiation B
VEGF	vascular endothelial growth factor
WNT	“wingless-related integration site” protein family
c-KIT	tyrosine-protein kinase KIT
c-MET	mesenchymal-epithelial transition factor
cAMP	cyclic adenosine monophosphate
iNOS-1	inducible nitric oxide synthase
iPLA2	calcium-independent phospholipase A2
mTOR	mammalian target of rapamycin
ox-LDL	oxidized LDL
p53	tumor protein p53
sFRP2	secreted frizzled-related protein 2
